# The Limpet controlled drug cabinet alarm and camera

**DOI:** 10.1186/cc13192

**Published:** 2014-03-17

**Authors:** M Mariyaselvam, D Pearson, P Moondi, P Young

**Affiliations:** 1 Queen Elizabeth Hospital NHS Trust, Kings Lynn, UK

## Introduction

The theft and tampering of controlled drugs (CDs) remains a prevalent patient safety issue. Sadly there are numerous reports of critical care staff stealing CDs for personal use or financial gain and notably there have been some cases where CDs have been substituted for other medications in order to delay detection of the theft. This creates both the hazard of medication errors and potentially exposes patients to opioid intoxicated healthcare workers. As most critical care staff have access to CDs, when drugs are found to be missing it can be difficult to identify the perpetrators. Therefore the implementation of a deterrent which also improves the methods of detection is warranted.

## Methods

The Limpet, a device which incorporates a proximity sensor and a camera unit, was installed within the CD cupboard of the critical care unit. Whenever the cupboard was accessed the date and time were recorded and a photograph was taken to identify the staff member. Mock thefts were subsequently undertaken by a designated staff member at random times. This allowed testing of the product to determine the number of times the ‘thief' was correctly identified.

## Results

Mock thefts were successfully accomplished on six occasions over a 4-week period. On each occasion the Limpet photographed the ‘thief' and recorded the date and time of access. Therefore, in the event of a real theft it would be possible to quickly and easily indentify the culprit.

## Conclusion

When CDs are missing it can be extremely stressful for the staff involved. Those who have access to CDs may feel unfairly scrutinised and the potential for false accusation exists. Investigating the theft of CDs is costly and usually involves pharmacists, managers and the police. Until the issue is resolved, potential suspects are usually suspended from work, leading to disruptions in patient care. The utility of the Limpet in modifying staff behaviour by reducing the number of occasions and the duration of time that open drug cupboards are left unattended, has previously been demonstrated [1]. By providing the facility to determine exactly who accessed each CD cupboard at which time, this initial study has shown the benefits of the Limpet as a tool for detecting theft. Therefore the installation of the Limpet mitigates the difficulties of investigating CD theft and is likely to prove an effective deterrent.
